# Book Reviews

**Published:** 1905-05

**Authors:** 


### BOOK REVIEWS.

The Modern Mastoid Operation. By Frederick Whiting, A.M., M.D., Professor of Otology, Cornell University Medical College. Royal Square octavo, pp. xiii.-353. Illustrated by 25 half-tone and 23 key plates made from original drawings. Philadelphia: P. Blakiston's Son & Company. 1905. (Half morocco, $6.00 net.)

We approach the notice writing of this book with a feeling akin to that inspired by viewing wondrous works of art, or nature, or reading of the marvelous achievements of great men,—explorers, warriors, artisans, engineers, inventors, and scientists. It unfolds to view and to thought one of the great advances in special surgery of the present day, and makes one feel proud of a profession that is accomplishing so much for the relief of suffering and the prolongation of life.

The author calls it a "little book" with a modesty that is refreshing. As a matter of fact it is one of the largest books yet printed, if judged by its importance, or by the benefit that will be derived from it,—speaking, of course, from the viewpoint of intelligent prophecy. Again, it is large considered in regard to its material size being, according to printing house parlance, a royal square octavo, with single columns measuring 4½ by 7⅜ inches each, containing approximately 320 words in number ten type. Finally, it is large if considered in relation to its illustrations. It contains 25 half-tones with an almost equal number of key or explanation plates made from original drawings. These engravings become at once the admiration of the artistic anatomist and the delight of the specialist who is in search of more information on mastoid disease.

Coming now to the text, after giving the history of the operation and describing the Schwartze method, the author proceeds to detail the mastoid operation as performed at his own clinics which he designates "the complete mastoid operation" and which was devised by himself, insofar as it differs from the commonly accepted procedure. The first difference characterising this operation is the tegumentary incision, which provides a flap approach to the mastoid apophysis. The length of the incisions will vary according to necessity. They may be lengthened on the one hand to meet special conditions, or shortened on the other, if the case is uncomplicated. A minute description and measurement of the incisions is given, indicating the average, or those most advantageous in the majority of instances.

A second difference between the author's operation and the Schwartze method, as modified by Gruening, consists of the removal of pneumatic spaces and diploic cells at the posterior root of the zygoma. If this is done in each and every instance, Whiting thinks the necessity of reoperating will be reduced to a minimum. He records two hundred and fifty consecutive complete mastoid operations with but a single reopening to induce healing, and that in a phthisical patient with reduced vital forces. Two hundred mastoid operations were made previously, eight per cent. of which required secondary operations. Under these circumstances the author is justified in claiming that his innovations are not only judicious but essential.

After discussing the pathology of suppurative mastoiditis, Whiting describes the preliminary preparations for the operation, then the tegumentary incisions and constructions of the flaps; next, the elevation of the periosteum and the retraction and reflection of the flaps; then the construction of the initial groove-shaped opening in the mastoid cortex; next, the construction of successive cortical grooves parallel with the initial groove; then the determination of the situation and dimensions of the antrum and the exposure of that cavity; next, the removal of the mastoid tip; then the removal of the mastoid cortex and cells overlying the sigmoid groove; the removal of the cells at the posterior root of the zygoma; the cleansing of the wound and the approximation and stitching of the flaps; the dressing of the wound and the application of the bandage; and, finally, the post-operative care of the mastoid wound. To each of these separate steps a chapter is devoted and each is also separately and superbly illustrated.

The last two chapters but one are concerned with the indications for the mastoid operation, including differential diagnosis; and the enumeration and description of the instruments required for the mastoid operation. A brief chapter is added giving conclusions, and a well prepared index closes the volume.

The author has given the most carefully prepared description and illustration of a single surgical procedure with which we are familiar. It is elaborately printed on number one book paper, with half-tones and drawings delineating the several details with remarkable precision,—the whole being a credit to American surgery and authorship. The perfection and beauty of the mechanical construction of the book entitle it to take rank as an *edition de luxe.*

Normal Histology and Microscopical Anatomy. By Jeremiah S. Ferguson, M. Sc., M. D., Instructor in Normal Histology, Cornell University Medical College, New York. Octavo, pp. 758. With 462 illustrations, many in colors. New York and London: D. Appleton & Company. 1905. (Price, $4.00).

The study of histology is now recognised as only second in importance to microscopical anatomy. As the latter deals with the structure of the organs, so the former is concerned with the finer structures of the tissues. The field contemplated by this treatise, inadequately covered though it has been in the past, is receiving the benefit of the experience of new observers with a frequency that justifies expectation that it will soon stand abreast of any of the advanced laboratory sciences.

An examination of this book, even by one little skilled in the science of histology, suggests the thought that it is a most excellent treatise; while to the laboratory instructor it will become apparent that no superior elaboration of the topic has been made by any American author. Ferguson's method is simple, his application is forceful and his conclusion is convincing. He does not give us theory, but facts; his teachings are not chimerical, but practical; and his work is not ephemeral, but lasting in character.

The advanced student of histology will discover in this work something which he has not seen before on almost all of the leading topics discussed; for example, the nerve tissue, the skin, the digestive system, the respiratory system, the reproductive organs and the glandular systems, one and all, appear in a new light, or at least possess new interest as dealt with by Ferguson.

Any notice of this book which fails to take cognisance of its illustrations does imperfect justice to one of its leading features. Of the four hundred and sixty-two figures, all in the text, two hundred and twenty-two are from original drawings and photomicrographs. These originals were prepared from microscopical specimens in actual use during laboratory instruction. They serve their intended purpose better than any similar drawings with which we are familiar.

We expect to see this treatise adopted as a textbook in all registered American medical colleges.

Blakiston's Quiz Compends. A Compend of the Diseases of the Eye and Refraction. By George M. Gould, Editor of American Medicine and Walter L. Pyle, Assistant Surgeon to Wills Eye Hospital, Philadelphia. Third edition, revised and corrected. Illustrated. Philadelphia: P. Blakiston's Son & Co. 1904. (Price, $1.00.)

The study of the eye and its diseases, including refraction, will be greatly facilitated by this compend, which is likely to attract the attention of medical students in particular. It contains also much information, arranged in convenient form, for the general practitioner who should always be conversant with these topics in order to send his patients to the proper specialist at the proper time. This edition has brought the subject forward to the present time through appropriate revision and correction.

PROGRESSIVE MEDICINE, Volume I., March, 1905. A Quarterly Digest of Advances, Discoveries and Improvements in the Medical and Surgical Science. Edited by HOBART AMORY HARE, M.D., Professor of Therapeutics and Materia Medica in the Jefferson Medical College of Philadelphia. Octavo, 298 pages, 10 engravings and a full-page plate. Philadelphia and New York: Lea Brothers & Company. (Per annum, in four cloth-bound volumes, $9.00; in paper binding, $6.00, carriage paid to any address.)

In this number we find surgery of the head, neck, and thorax by Charles H. Frazier; infectious diseases, including acute rheumatism, croupous pneumonia, and influenza by Robert B. Preble; the diseases of children by Floyd M. Crandall; laryngology and rhinology by Charles P. Grayson; and otology by Robert L. Randolph. The repairs of facial injuries and deformities furnishes an interesting chapter of modern surgery. The contribution of Professor Frazier here noted relating to this subject will be read with instructive interest by all medical men who deal with surgery in any manner, and especially by laryngological and rhinological surgeons.

The publishers very appropriately say of this class of literature in general, which of course applies to Progressive Medicine in particular, that "the enormous annual output of medical literature,—whether in the form of investigation into causes, pathology, symptoms, or therapy of disease,—is in such grotesque disproportion to the reading capacity of any one individual, the demand of some method of selection and of condensation becomes imperative."

No truer words ever have been said on this point and nothing more appropriate could be said in regard to the value of this particular series of condensed literature.

---

A Compend of the Practice of Medicine. By Daniel E. Hughes, M. D., formerly Demonstrator of Clinical Medicine in the Jefferson Medical College of Philadelphia. Seventh revised edition by Samuel Horton Brown, M. D., Assistant Dermatologist, Philadelphia Hospital. Duodecimo, pp. 779. Illustrated. Philadelphia: P. Blakiston's Son & Co. 1904. (Price, flexible leather, $2.50.)

An examination of this book shows that it is no longer a mere compend as when it began, but is in this new edition a digest of internal medicine, complete in material and perfect in form. Moreover, its typography is entirely new, it is increased by 137 pages, and twenty-seven illustrations have been added, the whole going to make up one of the most comprehensive works of its kind published in English. New material incorporated in this issue includes articles relating to general characteristics and classification of fevers, examination of the blood, sputum, and contents of the stomach; together with sections on urinalysis and physical diagnosis, as well as much other subject matter which has been added to render it a book of today. It is the exponent of the better class of compends and will continue to be a favorite.

---

Transactions of the American Gynecological Association. Vol. XXIX. Twenty-ninth annual meeting held at Boston, May 24-26, 1904. J. Riddle Goffe, M.D., Secretary. Philadelphia: William J. Dornan, Printer. 1904.

This sturdy society again offers its work for a year in most approved fashion. The scientific work of this body is not second to that of any special society, and this particular volume contains much material of important value. The traditions of the society are marked by a conservatism that is at once dignified and commendable. Sometimes this element leads to the verge of the danger line but not in this organisation. This volume constitutes a distinct addition to gynecological literature.

---

Pneumonia and Pneumococcus Infections. By Robert B. Preble, M.D., Professor of Medicine, Northwestern University. Duodecimo, pp. 211. Illustrated. Chicago: Cloyd J. Head & Co. 1905. (Price, $1.00.)

The author of this well written monograph explains that whenever the word pneumonia is used in the text, it means acute inflammation of the lungs with exudation in the alveoli, as a result of infection with the diplococcus pneumoniæ of Fraenkel-

Weichselbaum. In thus restricting the definition of pneumonia it makes the term correspond to our knowledge of the pathology of the disease as at present promulgated from the laboratory.

The bedside picture of pneumonia presented by this author is that recognised today by the scientific clinician, and it can be read with profit by juniors as well as seniors,—by clinical assistants and general practitioners of medicine. The great destructiveness of pneumonia renders it essential for physicians to study its causes, prevention, and treatment with a closer scrutiny.

---

Transactions of the American Surgical Association. Vol. XXII. 1904. Edited by Richard H. Harte, M.D., Recorder of the Association. Philadelphia: William J. Dornan, Printer. 1904.

One of the most remarkable surgical procedures, whether judged by the audacity of the operator or the results of the operation, is recorded in this book. We refer to a resection of the upper jaw for osteochondromyxosarcoma by Dr. S. J. Mixter, of Boston. The mass was large and the features of the patient were horribly distorted. Recovery was slow but complete and a year afterward the patient had gained about thirty pounds in weight. Dr. Mixter received the congratulations of his colleagues. The book is filled with interesting surgical literature. The president for this year is Dr. George Ben Johnston, of Richmond, and the meeting will be held in San Francisco, July 5, 6 and 7,—the week prior to the meeting of the American Medical Association at Portland.

